# Narrative Discourse in Young and Older Adults: Behavioral and NIRS Analyses

**DOI:** 10.3389/fnagi.2018.00069

**Published:** 2018-03-16

**Authors:** Charles-Olivier Martin, Stéphanie Pontbriand-Drolet, Valérie Daoust, Eric Yamga, Mahnoush Amiri, Lilian C. Hübner, Bernadette Ska

**Affiliations:** ^1^Faculty of Medicine, Université de Montréal, Montreal, QC, Canada; ^2^Centre de Recherche, Institut Universitaire de Gériatrie de Montréal, Montreal, QC, Canada; ^3^Génie Biomédical, École Polytechnique de Montréal, Montreal, QC, Canada; ^4^Departamento de Linguistica, Pontifícia Universidade Católica do Rio Grande do Sul (PUCRS), Porto Alegre, Brazil

**Keywords:** aging, discourse comprehension, cerebral plasticity, language, NIRS

## Abstract

Discourse comprehension is at the core of communication capabilities, making it an important component of elderly populations’ quality of life. The aim of this study is to evaluate changes in discourse comprehension and the underlying brain activity. Thirty-six participants read short stories and answered related probes in three conditions: micropropositions, macropropositions and situation models. Using near-infrared spectroscopy (NIRS), the variation in oxyhemoglobin (HbO_2_) and deoxyhemoglobin (HbR) concentrations was assessed throughout the task. The results revealed that the older adults performed with equivalent accuracy to the young ones at the macroproposition level of discourse comprehension, but were less accurate at the microproposition and situation model levels. Similar to what is described in the compensation-related utilization of neural circuits hypothesis (CRUNCH) model, older participants tended to have greater activation in the left dorsolateral prefrontal cortex while reading in all conditions. Although it did not enable them to perform similarly to younger participants in all conditions, this over-activation could be interpreted as a compensation mechanism.

## Introduction

Comprehension is a basic component of communication. Being able to extract the gist of discourse, while reading a text or listening to a speech, and relate it to our own knowledge of the world is essential for appropriate behavior and interaction in society. Thus, understanding the changes in discourse comprehension capacity and in the underlying brain changes during aging is crucial to maintain the elderly population’s quality of life.

The aim of this study is to evaluate changes in discourse comprehension and the underlying brain activity. The article is structured in four sections reflecting the way the study was organized in order to reach this aim: (1) the model allowing the discourse analysis on which most of studies are based; (2) results of studies on discourse comprehension, neural substrate and aging; (3) models of cerebral plasticity in aging; (4) near-infrared spectroscopy (NIRS) technique used to evaluate the cerebral activity during the reading task.

The construction-integration model (Kintsch and Van Dijk, 1978; Kintsch, 1988; Lebreton, 2012) describes comprehension as a progressive construction, through many cycles, in which successive propositional networks are elaborated and integrated into the networks built in prior cycles to form a cohesive whole. This model lists four levels of representation in the comprehension process that interact back and forth through the cycles of comprehension. Those levels will be described below.

First, the surface form refers to the linguistic components of discourse such as vocabulary and grammar without regard to its contents (Ska and Duong, 2005; Chesneau et al., 2007b).

Second, the textbase corresponds to the propositions of a text joined together in a structured, cohesive network (Kintsch and Van Dijk, 1978). Two types of propositions and structures can be distinguished in the first model developed by Kintsch: (a) Micropropositions and microstructure contain all the propositions of the discourse in a hierarchical network (Kintsch and Van Dijk, 1978; Lebreton, 2012). Thus, the microstructure comprises all the information and details of the discourse. (b) Macropropositions and macrostructure are derived from the microstructure by deleting the unneeded propositions and generalizing some groups of propositions, forming the gist of the discourse (Kintsch and Van Dijk, 1978). The macrostructure reduces and organizes the information in the textbase without losing the significance of the discourse (Denhière and Baudet, 1992).

Third, in the updated construction-integration model, the situation model tends to overlap the macrostructure. The situation model is a mental representation of the discourse integrating the individual’s knowledge (Kintsch, 1988; Lebreton, 2012). Unlike the textbase, the situation model does not represent the text itself but the subjects and events described in it (Radvansky et al., 2001). A situation model is a situation containing events, objects and characters delimited in time and space. An event taking place in a different time or space would be considered part of a new situation model (Radvansky and Dijkstra, 2007). The situation model results from inferencing capacity, which is essential to comprehension, because it associates with the text the necessary information from the knowledge of an individual and integrates the various pieces of information in the discourse (Perrig and Kintsch, 1985). In our study, the gist of information portion of the mental representation is classified as the macrostructure, and the inferences needed to properly build the mental representation are classified as the situation model.

Finally, the superstructure constitutes a framework for the other levels corresponding to the type of discourse presented (e.g., narrative or procedural). This framework allows one to anticipate and orient information processing depending on the type of discourse (Ska and Duong, 2005). This level will not be considered further here, because all texts in this study are narratives. Thus, this study will focus only on the textbase and the situation model.

The processes needed to accomplish discourse comprehension involve several cognitive functions and brain regions. Studies have shown that inhibition (Kintsch, 1988) and working memory (Radvansky and Copeland, 2004; Chesneau et al., 2007a) are involved at the textbase level, while inhibition (Radvansky, 1999b), long-term working memory (Ericsson and Kintsch, 1995) and semantic memory play a role at the situation model level (Radvansky and Dijkstra, 2007). However, the role of working memory in discourse comprehension is controversial. Some studies come to the conclusion that working memory does not influence the discourse comprehension (e.g., Evans et al., 2015) while others argue the opposite (Noh and Stine-Morrow, 2009; Borella et al., 2011; Payne and Stine-Morrow, 2017). The methodology and tasks used in these studies may explain these divergent results.

Although discourse comprehension depends mainly on the left hemisphere, most of the relevant processes need the contribution of both hemispheres (Perfetti and Frishkoff, 2008). Story comprehension entails a network of frontal, temporal and cingulate areas, which also are association areas (Mar, 2004). The anterior temporal lobes and left middle temporal gyrus could be involved in the semantic integration and interpretation of text in discourse as they are only activated while listening or reading meaningful texts or sentences (Mazoyer et al., 1993; Ferstl et al., 2008; Perfetti and Frishkoff, 2008; Bowman and Dennis, 2015; Zhuang et al., 2016). The prefrontal cortex, especially the superior dorsomedial prefrontal cortex, may be involved in the integration of one’s knowledge or when inferences from earlier parts of the discourse are needed, thus contributing to the maintenance of global coherence of texts (Schmalhofer and Perfetti, 2007; Perfetti and Frishkoff, 2008; Miotto et al., 2014; Bowman and Dennis, 2015).

Aging is known to have an impact on many cognitive functions, some of which, like working memory, are involved in discourse comprehension (Brébion, 2003; DeDe et al., 2004; Payne and Stine-Morrow, 2017). Among other things, elderly participants seem to rely more on the gist of the text and the situation model while performing comprehension tasks, while younger participants rely more on the textbase and microstructure (Radvansky et al., 2001; Ferstl, 2006; Radvansky and Dijkstra, 2007; Magliano et al., 2012; Davis et al., 2015; Zhuang et al., 2016). On the other hand, it seems that the characteristics of a text, especially its semantic load, highly influence the capacities of older adults to recall the microstructure and macrostructure. In fact, older adults have more problems recalling the microstructure of texts containing a lot of propositions (high semantic load) than texts with few propositions (low semantic load). Conversely, they have more problems recalling the macrostructure of texts with a low semantic load than texts with a high semantic load (Ferstl, 2006; Chesneau et al., 2007a). These results highlight the complex influence of working memory on the changes in discourse comprehension during aging, as it seems to be easier to handle and extract the essential information of a text when it has a high semantic load, even if each propositions become harder to recall in itself.

Several models have sought to explain the brain plasticity mechanisms that apply during aging in relation to cognitive decline. One model in particular is relevant to our study.

The compensation-related utilization of neural circuits hypothesis (CRUNCH) specifies that the over-activation of brain regions often observed in elderly participants is a compensation mechanism (Reuter-Lorenz and Cappell, 2008; Fu et al., 2017). This over-activation may entail activation of more regions than in younger people or greater activation of the region normally dedicated to the task. Sometime, an interhemispheric or an intrahemispheric reorganization seems to take place such as, respectively, in the HAROLD (Cabeza, 2002) and PASA models (Davis et al., 2008). Although the goal of those mechanisms is to maintain performance, they are not always sufficient (Reuter-Lorenz and Cappell, 2008). The dorsolateral prefrontal cortex often plays a compensatory role (Reuter-Lorenz and Cappell, 2008; Cappell et al., 2010).

Many studies investigated the influence of aging on discourse comprehension (e.g., Stine-Morrow et al., 2001, 2008; De Beni et al., 2003; Hannon and Daneman, 2009; McGinnis, 2009; Noh and Stine-Morrow, 2009; Wright et al., 2011; Mund et al., 2012). Other studies used cerebral imagery approaches to elicit neural contribution to language and semantic treatment (e.g., Cahana-Amitay and Albert, 2014; Shafto, 2015; Wang et al., 2015; Cohen et al., 2016). One previous study combined both and investigated cerebral plasticity in a discourse comprehension task. It described both an intrahemispheric and an interhemispheric reorganization in the elderly group (Scherer et al., 2012). Nonetheless, that study only collected data from the posterior part of the frontal lobes and a part of the temporal lobes and used a small sample size. Another study (Martin et al., 2017) compared high and low educated old participants in a task of discourse comprehension and did not find differences between the two groups on the behavioral and the cerebral activity. Thus, the global picture of cerebral plasticity in discourse comprehension is still incomplete.

NIRS technique uses near-infrared light to examine changes in the concentration of susceptible chromophores in this spectral window being oxyhemoglobin (HbO_2_) and deoxyhemoglobin (HbR; Jöbsis, 1977). Since those chromophores partially absorb near-infrared light, in the range of 650–950 nm, a change in their concentration will alter the intensity of light passing through the biological tissue (Strangman et al., 2002; Kocsis et al., 2006; Gratton and Fabiani, 2007). Brain activity induces a local hemodynamic response, which is characterized by an increase in HbO_2_ concentration coupled with a decrease in HbR concentration and is followed by a return to normal (Chance et al., 1993; Hoshi, 2007; Racz et al., 2017). This light-medium interaction allows NIRS to be used for assessing hemodynamic changes and thus estimating local brain activity as a result.

This technique is known to have a temporal resolution around 10 ms and a spatial resolution of 1–3 cm, which gives a better temporal resolution but poorer spatial resolution comparing to the functional magnetic resonance imaging (fMRI) and better spatial resolution but poorer temporal resolution than electroencephalography (EEG; Strangman et al., 2002; Rodden and Stemmer, 2008). It’s totally non-invasive nature facilitates studies with babies, young children and elderly people (Strangman et al., 2002). For purposes of our study, NIRS is particularly useful because it allows one to create a more natural environment for reading, namely a sitting position without background noise, unlike fMRI; therefore, it is less disturbing for participants, especially elderly people (Hoshi, 2007).

The aim of this study was to use NIRS technique to evaluate the effect of aging on different levels of discourse comprehension and on the brain activity related to those levels. Another goal of this study was to set the bases concerning the evolution of discourse comprehension in normal and optimal aging with a view to future studies investigating differences within the aging population. Two main hypotheses were formulated. First, at the behavioral level, the older participants should be less accurate at the microproposition level than the younger participants, but equally (or almost equally) accurate at the macroproposition and situation model level. Second, consistent with the CRUNCH plasticity model discussed above, the older participants would show greater hemodynamic response in the prefrontal cortex. Differences between the three levels of discourse comprehension studied were not expected on the aspect of hemodynamic responses because of the simultaneous processing of those comprehension levels.

## Materials and Methods

This project was approved by the *Comité mixte d’éthique de la recherche du Regroupement Neuroimagerie Québec* (Joint Committee on Research Ethics of the Quebec Neuroimaging group). All subjects gave written informed consent in accordance with the Declaration of Helsinki.

### Participants

Thirty-six adults participated in this study: 18 older adults, from 66 to 78 years old, and 18 younger adults, from 20 years to 27 years old. All had university degrees (15–21 years of education). All were right handed, as assessed by the Edinburgh Handedness Inventory (Oldfield, 1971), with a score of 80 or higher; had French as their mother tongue; and had normal or corrected vision, as assessed by the Near Vision Test Card (Snellen, 1862). Their vocabulary level was evaluated with the Mill Hill Vocabulary Scale (Deltour, 1993). Participants did not have dyslexia, diabetes, psychiatric or neurological impairments, or a history of alcoholism or drug addiction.

All older participants had a score of 26 or more on the Montreal Cognitive Assessment (MoCA; Nasreddine et al., 2005). All participants underwent a short neuropsychological testing to assess their cognitive functions, including working memory (Digit Span test; Wechsler, 1991), short-term and long-term memory (Rey Auditory Verbal Learning Test; Rey, 1970), attention (Trail Making Test, parts A and B; Reitan and Wolfson, 1985), inhibitory control (Stroop Victoria Test; Stroop, 1935), mental flexibility (Wisconsin Card Sorting Test, 64-card version; Nelson, 1976) and processing speed (Digit Symbol; Wechsler, 1991); see the Appendix Table 1 for details.

### Stimuli

**Figure 1:**
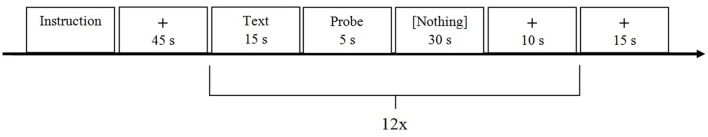
Timeline and task design for each block of stimuli. Participants had 15 s to read the text. Then a probe was displayed for 5 s, as soon as the probe appeared, participants had 10 s to answer as fast and accurately as they could. When the probe disappeared, blank screen was displayed for 30 s (Nothing). Ten seconds before the next text presentation, a cross appeared to notify the participants of the upcoming text. This sequence was repeated 12 times.

The task is composed of 36 short stories and probe questions created by Scherer et al. (2012). The story-probe combinations were divided into three blocks evaluating the different components of the discourse comprehension model: micropropositions, macropropositions and situation model. The micropropositions block refers to the raw textual information, the macropropositions block refers to the extraction of the gist of texts via the macrorules (deletion, generalization and construction) and the situation model blocks refer to the capacities to inference a conclusion combining one’s knowledge and information from the text. Participants read a short story on a computer and, immediately afterward, they pressed a button to indicate whether the presented probe was true or false, according to the story they just had read. True and false answers were balanced in each condition and the order of block presentation was counterbalanced between participants. None of the stories contained metaphors, irony or indirect language. Short stories were chosen to decrease the influence of working memory on discourse comprehension by reducing the information load. All stories were built to be equivalent regarding number of words, syllables, letters, sentences and propositions. All stories were also equivalent regarding syntactic complexity. The timeline and task design are presented in Figure 1.

Examples of three short narratives with their corresponding probes.

#### Microproposition Level

Text: Michaël est propriétaire d’une voiture verte depuis douze ans. Il l’aime bien et l’appelle Pierrette. La voiture de Michaël est très vieille et tombe en panne. Michaël est triste parce qu’il va devoir acheter une autre voiture.

*Michaël has owned a green car for 12 years. He loves it and calls it Pierrette. Michaël’s car is very old and breaks down. Michaël is sad because he will need to buy another car*.

Probe: La vieille voiture de Michaël est de couleur bleue. (F)

Michaël’s old car is blue. (F)

#### Macroproposition Level

Text: Joanne a l’habitude d’être malade durant ses voyages, mais aujourd’hui elle se sent bien. Soudain, la plus jeune de ses filles tombe. Heureusement, un autre touriste la ramène à bord en la tirant par son gilet de sauvetage.

*Joanne usually gets sick when traveling, but today she feels well. Suddenly, her youngest daughter falls. Fortunately, another tourist pulls her back on board by grabbing her life jacket*.

Probe: Joanne et sa fille sont des touristes en croisière sur un bateau. (V)

Joanne and her daughter are tourists traveling on a boat. (T)

#### Situation Model Level

Text: Marie ne parle pas couramment le français. Elle a lu une offre d’emploi de réceptionniste dans un hôtel de Montréal. Elle va à l’entrevue mais sa candidature n’est pas retenue. Marie décide de prendre des cours de français.

*Marie does not speak French fluently. She has read a posting for a job as a receptionist in a Montreal hotel. She goes for the interview, but her application is not accepted. Marie decides to take French courses*.

Probe: Marie étudiera le français parce qu’elle adore cette langue. (F)

Marie is going to study French because she loves the language. (F)

### Procedure

During the task, participants were seated on a chair in front of a computer while wearing a NIRS helmet. Their index fingers were placed on two buttons, one with a green “V” (for *vrai* in French, meaning “true”) and one with a red “F” (for *faux* in French, meaning “false”). The locations of the buttons were counterbalanced among the participants. During the task, they were asked to read silently and to avoid speaking or moving. After receiving the instructions, the participants performed a practice task composed of three stories, each followed by a probe. Then, they performed the three blocks (duration of 780 s each) with a few minutes’ pause after each block. Participants had 15 s to read each text, after which they had 10 s maximum to read and answer the probe about the preceding text. While no instructions were given about reading speed, participants were asked to answer as quickly as possible when the probe appears on the screen. All participants were able to answer in the given time.

### Data Acquisition

**Figure 2:**
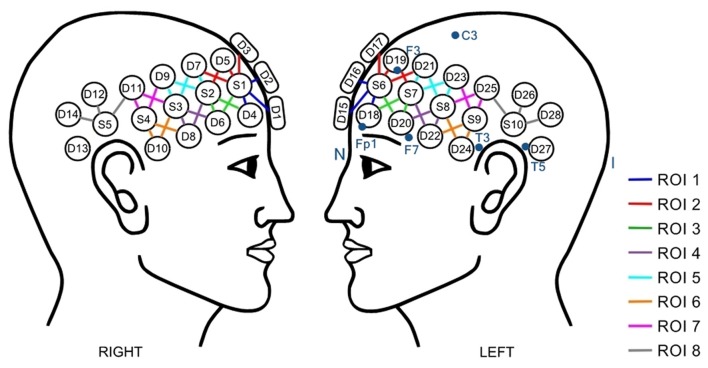
Positions of sources (S), detectors (D) and regions of interest (ROI).

Behavioral data were recorded using E-Prime2 program^1^. Functional NIRS data were acquired using a TECHEN Continuous Wave systems model CW6. Ten sources and 28 detectors were located on each participant’s head, according to the language-related brain regions, for a total of 58 optical channels. Each source consisted of two lasers emitting light at 690 nm and 830 nm. After the acquisition, channels were grouped in 16 regions of interest (ROIs) evenly distributed on each brain hemisphere. The helmet was placed on each participant’s head based on the 10-20 standard (Jasper, 1958) as a reference. The helmet design and the position of sources, detectors, and ROIs are presented in Figure 2. To insure reliability, for each participant, nasion-inion distance was measured and the helmet was placed at 10% of that distance from the nasion. Other points of reference were measured to ensure good positioning of the helmet (see Figure 2). Anatomical MRI from a previous study (Amiri et al., 2014) with the same helmet allowed us to estimate the Brodmann’s Area covered by each ROI (see Table 1). NIRS acquisition was performed throughout each block of the task and trigger points were set at the beginning of each story and probes to dissociate those segments in the analyses.

**Table 1:** Estimation of the Brodmann’s area covered by each region of interest (ROI).

Region of interest	Estimated corresponding Brodmann’s area
ROI 1	46 – Dorsolateral prefrontal cortex
	10 – Frontopolar area
ROI 2	9 – Dorsolateral prefrontal cortex
	44 – Pars opercularis, part of Broca’s area
ROI 3	45 – Pars triangularis, part of Broca’s area
ROI 4	6 – Premotor and supplementary motor cortex
	48 – Retrosubicular area
ROI 5	6 – Premotor and supplementary motor cortex
	43 – Subcentral area
ROI 6	21 – Middle temporal gyrus
ROI 7	22 – Superior temporal gyrus
	40 – Supramarginal gyrus, part of Wernicke’s area
ROI 8	37 – Fusiform gyrus

### Data Analysis

NIRS data were processed, normalized and analyzed using HomER2^2^—that is acronym for Hemodynamic optically measured Evoked Response—an open-source data analysis toolbox with GUI interface. HomER2 provides users with tools to estimate the concentration changes for HbO_2_ and HbR from the change in light intensity recovered by detectors with NIRS (Huppert et al., 2009). Channels S4-D12, S5-D10, S5-D13, S9-D26, S10-D24 and S10-D27 were recorded but excluded from the analysis because of a poor signal across most participants. Statistical analyses of the behavioral and NIRS data (mean amplitude of hemoglobin concentration’s variation) were performed using SPSS software^3^. Data distributions were normal. Thus, independent sample Student *t*-tests were used to compare the two groups at the behavioral level. As the two independent variables (level of discourse comprehension and age) studied here were not related, one-way analysis of variance (ANOVAs) with a Bonferroni correction were used to compare mean hemoglobin (Hb) changes between each condition within groups for both parts of the task. Independent sample Student *t*-tests were used to compare mean Hb changes in each ROI between the two groups.

## Results

### Behavioral Data

The sociodemographic characteristics of both groups are presented in Table 2. Younger and older groups differed only in their age, since no significant differences were observed in terms of education and vocabulary levels.

**Table 2:** Sociodemographic characteristics of the groups.

Characteristics	Younger	Older	*t*-value	*P*-value
	(*n* = 18)	(*n* = 18)
	Mean (SD)	Mean (SD)
Age	23.56 (1.82)	72.33 (3.73)	−49.895	0.000
Gender (Male/Female)	7/11	6/12	n/a	n/a
Education	16.78 (1.11)	16.67 (1.97)	0.208	0.836
Mill Hill vocabulary test	25.28 (3.30)	27.50 (4.61)	−1.663	0.105

*Significant level was set at p ≤ 0.05. SD, Standard Deviation*.

**Figure 3:**
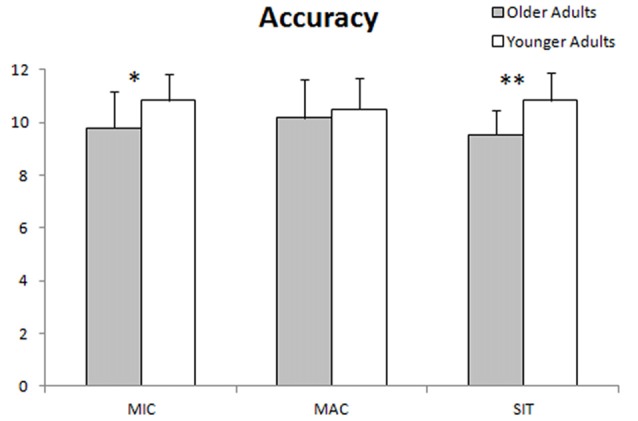
Accuracy in each condition (MIC, micropropositions; MAC, macropropositions; SIT, situation model). Significant level was set at *p ≤ 0.05; **p ≤ 0.01.

**Figure 4:**
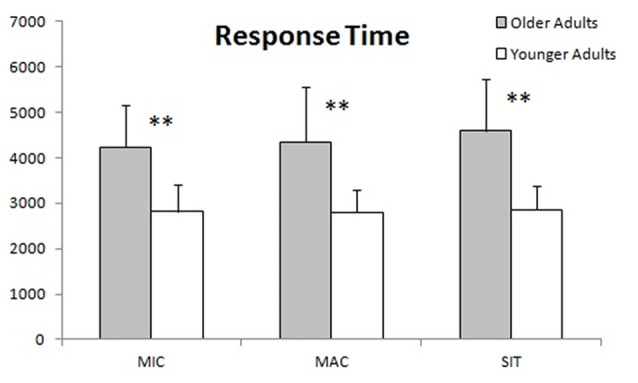
Response times (ms) in each condition (MIC, micropropositions; MAC, macropropositions; SIT, situation model). Significant level was set at *p ≤ 0.05; **p ≤ 0.01.

Numbers of correct answers for each condition in the reading task are presented in Figure 3. The younger group produced significantly more correct answers in the microproposition and situation model conditions than the older group. No significant difference was found in the macroproposition condition between the two groups. The results about microproposition and macroproposition conditions are in accordance with studies revealing that elderly participants perform better on what the text is *about* than its structure and contain (Radvansky et al., 2001; Radvansky and Dijkstra, 2007). The result about the situation model is a little more surprising, but may be congruent with short texts not allowing to properly build a situation model. Figure 4 shows response times; it reveals that the younger group responded significantly faster than the older group to the probes in all conditions. This result is probably the consequence of processing speed declines in aging (Salthouse, 1993).

### NIRS Data

**Figure 5:**
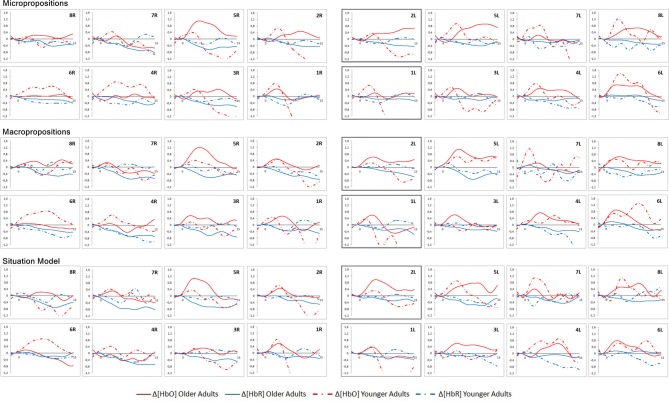
Grand-averaged waveforms of oxyhemoglobin (HbO_2_) and deoxyhemoglobin (HbR) concentrations during the reading portion of the task (15 s). Variation in hemoglobin concentration (μM) as a function of time (s). ROI with significant differences are highlighted in gray. R, Right; L, Left.

The grand-averaged waveforms of HbO_2_ and HbR concentrations during the reading part of the task are shown in Figure 5 for both groups in the three conditions.

No significant differences in HbO_2_ and HbR concentration variations were found between conditions in the within-group analysis (one-way ANOVA) during the reading portion of the task in either group. Those results were expected since brain activity while reading should stay constant and not be influenced by conditions. Student *t*-tests were performed for the between-group analysis. Significant differences between the two groups were found in ROI 1L and 2L: the older group showed a greater and more sustained increase in HbO_2_ in all three conditions while reading the texts. Significant differences were observed for region 1L, in the microproposition (*p* = 0.034) and macroproposition conditions (*p* = 0.028), with an almost significant difference in the situation model condition (*p* = 0.060). For region 2L, significant differences were seen in the microproposition (*p* = 0.021), macroproposition (*p* = 0.043) and situation model conditions (*p* = 0.015). These regions correspond to the left dorsolateral prefrontal cortex, the left frontopolar area and part of Broca’s area. This region is known to often play a role in compensation mechanisms (Reuter-Lorenz and Cappell, 2008).

**Figure 6:**
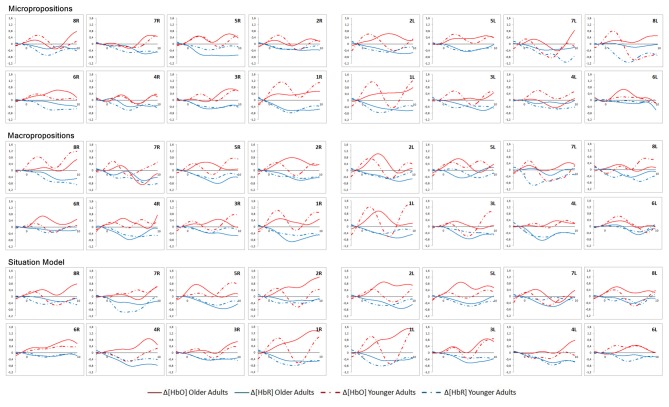
Grand-averaged waveforms of HbO_2_ and HbR concentrations while participants answered the probes. Variation (10 s) in hemoglobin concentration (μM) as a function of time (s). R, Right; L, Left.

The grand-averaged waveforms of HbO_2_ and HbR concentrations while participants answered the probe questions are presented in Figure 6 for both groups in the three conditions. No significant differences in HbO_2_ and HbR concentration variations were found between conditions in the intragroup analysis (one-way ANOVA) during the time allowed to answer the probe in either group. As well, no significant differences in HbO_2_ and HbR concentration variations were found between the two groups (Student *t*-test) while they were answering the probe questions.

## Discussion

Discourse is a highly elaborative linguistic ability (Ska et al., 2004). Consequently, it depends on several interrelated cognitive functions (Borella et al., 2011) and age related declines might be a consequence of deficits or modifications of a number of component processes rather than on specific processes (Hannon and Daneman, 2009). Moreover, the neural substrate is a complex network of frontal, temporal and cingulated areas (Mar, 2004).

### Differences in Task Accuracy

In order to reduce the influence of working memory on the older group’s performance, short texts and short waiting periods between the texts and the probes were used. Nevertheless, the older group still performed less accurately than the younger group in the microproposition condition. The older adults managed to maintain equivalent accuracy to the younger group in the macroproposition (gist) condition. Similar results had been reported in the past in studies using longer texts and longer periods between reading and questions (Radvansky et al., 2001; Radvansky and Dijkstra, 2007; Davis et al., 2015). According to those studies, elderly participants perform better and rely more on what the text is about (macrostructure and situation model) than what it is (surface form and micropropositions). Our study tends to confirm those findings at least regarding the microstructure and macrostructure. The shorter texts in our study may explain the differences with those studies on the situation model condition, and this will be addressed in the following section.

Since the influence of working memory was minimized, the fact that the older adults still performed worse at the microstructural level may have been because, in general, they relied less on that component of comprehension. It is possible that older adults read more efficiently and set aside information they do not need more quickly, emphasizing the gist of the story instead of assimilating the whole text. By doing this, they keep a smaller propositional network active, which is easier to process through the different cycles of comprehension. This network is enough to build the situation model but does not allow them to retain all the details of a text. This is also in line with the better ability of younger participants to recall the verbatim of texts, especially when the time period allowed for reading is short, as they are able to correctly recall a higher percentage of propositions from a text than older participants (Morrow et al., 1992). On the other hand, an inhibitory deficit in older adults may explain a difficulty to reject false but probable information especially since their attention is oriented to semantic information neglecting details (Hannon and Daneman, 2009; Wright et al., 2011).

Another potential explanation arises from the fact that the younger group was mostly composed of individual who had stopped attending school less than 5 years before participating in the study. Students tend to try to retain most of the details of a text while preparing for exams and they may maintain that habit even afterward while reading for a test. This type of reading is unnecessarily tiring and resource-demanding, because retaining every detail is not useful for overall comprehension. Thus, this habit is likely to gradually disappear when an individual has not had to study for a long time. This interpretation may also explain why younger participants seem to rely more on the microstructural level even in studies with longer texts (Radvansky et al., 2001).

Because the macroproposition block covers the gist of the text and the generalization of propositions, and because it is harder to evaluate change in the spatiotemporal framework within short texts, the situation model block covers mainly the integration and inferences of knowledge aspects of the model. Integration refers to the necessity to combine two or more pieces of information, and inference refers to the need to derive implications from the information presented (Sommers et al., 2011). Although the construction of the situation model is known to be preserved in aging (Radvansky et al., 2001; Radvansky and Dijkstra, 2007), the older adults responded less accurately in this condition than the younger ones. This difference may have arisen because this study focuses more on the integration and inference of knowledge aspects. Those types of questions were reported to be harder to answer than informal questions (and inferences are harder than integration) in a study of listening comprehension (Sommers et al., 2011). However, Sommers et al. (2011) also reported that the rate of age-related decline does not differ for the three types of questions.

Moreover, McGinnis (2009) reported that older adults generate more inferences while reading and that inferential processing may be less sensitive to cognitive decline. This is consistent with the hypothesis that older adults rely more on their prior knowledge in reading comprehension to compensate for the age-related decline in resource allocation (Maury et al., 2010). In this case, to explain the unexpectedly poorer performance of the older group in the situation model condition in our study, it may be that very short texts are too difficult because they do not provide enough time and redundancy of propositions to properly build a situation model. In that context, older adults may not have enough material to build a good connection with their prior knowledge. Since the situation model was not consolidated, the older participants found it more difficult to produce the inferences they needed to answer the probes. Maybe they would even have benefited from more time between the reading of the texts and the presentation of the probes to consolidate what they had just read in a better situation model with the proper inferences. In fact, the decline of processing speed in aging is well documented and can interfere with working memory and executive functions during discourse comprehension (Borella et al., 2011).

The older adults responded significantly slower than the younger ones in all three conditions. This result was expected since many studies report that processing speed declines in aging (e.g., Salthouse, 1993, 1996; Rabbitt et al., 2007; Feld and Sommers, 2009). Building the microstructural network, extracting the macrostructure (gist) and updating the situation model require a lot of cycles of processing by different types of memory, as well as inhibition (Radvansky, 1999a; Chesneau et al., 2007a). Thus, a decline in processing speed in the older group may also explain the differences in accuracy. This assumption is especially likely at the microstructural level, where the network grows more and more as the person reads, making it more difficult to process. Since the macrostructure is an extract of the essential components of the microstructure, it is easier to process, with fewer propositions to carry from one cycle to the next, and therefore may be less affected by the decrease in processing speed observed in aging.

### Constants and Changes in Hemodynamic Responses in Aging

The older and younger groups showed no significant differences in HbO_2_ and HbR concentration changes between the three conditions while reading and while answering the probe questions. Those results were expected since participants were not aware of the type of questions they would get after reading and answering involves retrieving information more than it depends on comprehension, which was done earlier in the process. Moreover, the levels of discourse comprehension are interconnected, simultaneous and self-directed while reading, making them nearly impossible to differentiate neuroanatomically.

No differences are observed in the temporal regions associated with discourse comprehension. In fact, while reading, both groups showed a substantial hemodynamic response in the left middle temporal gyrus (ROI 6L), which plays a role in the integration and interpretation of texts (Ferstl et al., 2008). Both groups also showed hemodynamic responses in the premotor and supplementary motor cortex (ROI 5, mainly in the left hemisphere). These regions are known to play an important role in reading aloud (Fiez and Petersen, 1998). While a person is reading aloud, some of the activation of the supplementary motor cortex is linked to the encoding of word form information, which may also be present during silent reading (Alario et al., 2006). The supplementary motor cortex is also activated when the retrieval of verbs from memory is necessary (Wise et al., 1991). The premotor cortex is also known to be activated while reading action words silently (Hauk and Pulvermüller, 2004). Hemodynamic responses were also present, in both groups, in the left fusiform gyrus (ROI 8L), which is associated with visual word recognition and is systematically activated during reading (McCandliss et al., 2003; Dehaene and Cohen, 2011).

No differences between groups were observed in the right hemisphere. The right hemisphere is mainly associated with prosodic and pragmatic processing (Ferré et al., 2011), which was avoided in this task. Thus, a task that emphasizes those aspects of discourse comprehension may reveal a greater difference between younger and older participants. Still, both groups demonstrated right frontal region activation during the reading part of the task, which could be linked to the integration of the propositional network built while reading (Robertson et al., 2000).

Significant differences between the younger and older groups were seen mainly in the left dorsolateral prefrontal cortex, part of the left frontopolar area and part of Broca’s area (ROI 1L and 2L) while reading the texts. Older adults sustained greater and longer hemodynamic responses. This difference may be due to the longer time needed to read the text or a greater need for attentional resources. It also probably indicates an attentional effort in semantic processing (Zhuang et al., 2016). The prefrontal cortex as a whole is known to be a major association area, having a pivotal role in the synthesis of the multifaceted information generated by the brain (Kandel et al., 2000). Even in normal brain aging, functional connectivity has been shown to decrease leading to restructured network topology and associated behavioral alterations which impact the dynamic and structure of the global network of the brain (Ferreira and Busatto, 2013). Thus, the over-activation of the left dorsolateral prefrontal cortex may be the results of this restructuration occurring in aging. Furthermore, the left dorsolateral prefrontal cortex had already been highlighted as a potential modulator of discourse comprehension. This region was observed to be more active in older participants who had good reading habits, but less active when these participants answered questions about their reading (Martin et al., 2012). This over-activation may reflect compensatory strategies in older adults, who possibly recruit more attentional resources while reading to maximize information encoding or make more efforts to properly integrate information. More studies on the subject would be necessary to confirm those results and investigated the potential modular role of the left dorsolateral prefrontal cortex in discourse comprehension.

Despite differences in accuracy and in response times, the older and younger adults showed no significant differences in any brain regions while answering the probe questions. Nevertheless, the younger participants showed short hemodynamic responses in the dorsolateral prefrontal cortex bilaterally while the older participants showed longer ones. This is certainly linked to the differences in response times discussed previously. Slower processes may generate more sustained hemodynamic responses, reflecting the time needed to perform the task.

### Discourse Comprehension and Models of Aging

Given that discourse comprehension is a complex but common task, it is interesting to see whether some well-known aging models may shed light on our results.

The greater brain activity described in the dorsolateral prefrontal cortex in the older adults is in line with the CRUNCH model which describes a role for that particular region in compensation mechanisms (Reuter-Lorenz and Cappell, 2008). Thus, the greater activation observed in our study may be a sign of a compensatory mechanism in the older group to achieve good comprehension while reading the texts. The CRUNCH model also states that over-activation is not always adequate to maintain performance in aging, which was the case in two out of the three conditions in this study.

Our study does not contradict an intrahemispheric reorganization like the one described in the PASA model (Davis et al., 2008) since there was greater activation in the anterior regions, but there was no decrease in activation in the posterior region. Since our study did not cover the whole posterior brain, it is not possible to rule out the existence of PASA phenomena in discourse comprehension.

It is also impossible to draw any conclusions concerning an interhemispheric reorganization like the one described in the HAROLD model (Cabeza, 2002). In fact, both older and younger adults showed signs of bilateralism while executing this comprehension task. This is not surprising, since discourse comprehension is a complex task involving many cognitive processes.

The present study does not replicate the previous finding of an interhemispheric and intrahemispheric reorganization in older adults reported by a study using a similar task by Scherer et al. (2012). That study used a helmet device covering only the posterior part of the frontal lobes and a part of the temporal lobes in a small sample of participants. In our study, even if a greater activation was observed in the anterior regions, no decrease in activation in the posterior regions was found. Furthermore, in our samples, both older and younger adults showed signs of bilateralism while executing the task which reflects the complexity of the cognitive processes involved.

### Future Considerations and Limitations

Number of texts and probes was one of the main limitations caused by neuroimaging techniques which required a relatively long cool-down between each trial. The new data presented here could help resolve this limitation. Indeed, the absence of difference between conditions on the hemoglobin variation level will prompt to develop a task with less texts, but more probes to answer.

This study compared two groups with university-level education. Older adults with a high education level are known to be generally less affected by cognitive decline. Less homogeneous groups could reveal more differences and lead to a better understanding of discourse comprehension in the elderly population. Varying the length of the texts and the level of difficulty could also provide interesting data to help gain a better understanding of older adults’ capacity for inferencing and building a situation model.

Another limit was that a task with short texts was used to help to find a balance between the needed parameters for the discourse comprehension’s capabilities and the many restraints of neuroimaging and NIRS technique. Short texts were also chosen to decrease the influence of working memory on discourse comprehension by reducing the information load. Even if the task helped to highlight differences at the microstructure and situation model levels between young and older participants, it was probably a little bit too easy for the younger participants given that most of them got a really high accuracy. Thereby, this task would not be suited for a study with only a heterogeneous group of young participants as it would not permit to differentiate strength and shortcomings between them. Long texts and short texts also seem to be different in terms of capacities to recall information, since older adults show more difficulties to recall situation model information than expected in short texts. Our comprehension of this phenomenon would benefit from further investigation.

Discourse comprehension, and its changes during aging, is a vitally important subject due to its role in preserving quality of life. Future studies should look at these changes in a more realistic task such as reading the newspaper or engaging in conversation.

## Conclusion

This study demonstrated that while older adults find it harder to recall the microstructure (details) or the situation model (inference) of a short text, they still manage to properly recall the macrostructure (gist of discourse). In accordance with the CRUNCH model, older adults show significantly greater activation in the left dorsolateral prefrontal cortex while reading texts in all conditions, although the increased activation was not sufficient to maintain the same accuracy as younger participants in two of the conditions. Thus, this greater activation in the left dorsolateral prefrontal cortex while reading is possibly link to a compensatory mechanism by the older adults. However, more studies are necessary to conclude on that point.

## Author Contributions

C-OM is the main author of this article and he was responsible of the protocol, data collection and analysis and mansucript’s redaction. SP-D contributed to data analysis. VD and EY contributed to recruitment and data collection. MA was the ressource person regarding NIRS technique and NIRS data treatment. LCH developped a prior version of the task used in this study. BS supervised this project as a thesis director. All authors approved the final version of this manuscript.

## Conflict of Interest Statement

The authors declare that the research was conducted in the absence of any commercial or financial relationships that could be construed as a potential conflict of interest.

## Footnotes

^1^http://www.pstnet.com/eprime.cfm

^2^http://www.nmr.mgh.harvard.edu/PMI/resources/homer2/home.htm

^3^http://www-01.ibm.com/software/analytics/spss/

## Appendix

**Table 1:** Neuropsychological characteristics of the groups.

Characteristics	Younger	Older	*t*-value	*P*-value
	(*n* = 18)	(*n* = 18)
	Mean (SD)	Mean (SD)
Edinburgh index	>80%	>80%	n/a	n/a
Near vision test card	Perfect 1.2 m	Perfect 1.2 m	n/a	n/a
MoCA	n/a	28.11 (1.32)	n/a	n/a
RAVLT				
Encoding	60.56 (5.40)	49.83 (7.28)	5.018	0.000
Delayed recall	13.89 (1.13)	11.00 (3.74)	3.135	0.005
Stroop victoria				
Dot—Time	10.57 (1.12)	12.62 (2.32)	−3.381	0.002
Word—Time	12.04 (1.48)	16.77 (3.89)	−4.831	0.000
Color—Time	16.48 (3.42)	27.57 (13.43)	−3.396	0.002
Trail making test				
A—Time	17.51 (4.21)	34.12 (8.22)	−7.634	0.000
B—Time	44.22 (13.77)	80.88 (25.77)	−5.325	0.000
B–A Time	26.70 (10.78)	46.76 (24.84)	−3.143	0.005
Digit span				
Forward	11.44 (1.85)	9.83 (2.41)	2.250	0.031
Backward	8.72 (2.02)	6.89 (2.52)	2.408	0.022
Wisconsin card sorting task				
Categories	4.56 (0.62)	3.50 (1.20)	3.319	0.003
Errors	10.06 (3.10)	14.39 (4.53)	−3.353	0.002
Digit symbol				
After 1 min	47.22 (4.85)	31.11 (6.25)	r8.645	0.000
After 2 min	96.28 (9.02)	63.22 (14.29)	8.302	0.000
2 min – 1 min	49.06 (5.10)	32.11 (8.42)	7.304	0.000
Matching	16.06 (2.67)	12.83 (2.75)	3.569	0.001
Free recall	8.39 (0.70)	7.50 (0.86)	3.411	0.002

*SD, Standard Deviation; MoCA, Montreal Cognitive Assessment; RAVLT, Rey Auditory Verbal Learning Test*.
